# Multi-center nationwide study on pediatric psychiatric inpatients 2000–2018: length of stay, recurrent hospitalization, functioning level, suicidality, violence and diagnostic profiles

**DOI:** 10.1007/s00787-021-01898-0

**Published:** 2021-11-22

**Authors:** Kim Kronström, Elina Tiiri, Miika Vuori, Heikki Ellilä, Anne Kaljonen, Andre Sourander

**Affiliations:** 1 Department of Adolescent Psychiatry, Turku University Hospital, Hospital District of Southwest Finland, Turku, Finland; 2 Research Centre for Child Psychiatry, University of Turku, Turku, Finland; 3 Department of Child Psychiatry, Turku University Hospital, Hospital District of Southwest Finland, Turku, Finland; 4 Master School, Faculty of Health and Wellbeing, Turku University of Applied Sciences, Turku, Finland

**Keywords:** Child and adolescent psychiatry, Diagnoses, General functioning, Inpatient treatment, Time-trend study

## Abstract

Inpatient treatment is an important part of child and adolescent psychiatric (CAP) care. This nationwide study explores the changes in length of stay, recurrent hospitalization, functioning level, suicidality, violence and diagnoses of CAP inpatients in Finland in 2000, 2011 and 2018. We obtained comprehensive questionnaire data on each study year from 93 to 95% of all CAP wards in Finland. We split the 1276 inpatients into two age groups in each study year: under 13 and 13–18. The median length of stay in inpatient treatment decreased from 82.0 days in 2000 to 20.5 days in 2018 (*p* < 0.001) and recurrent hospitalization increased from 38 to 46%. General functioning, which was evaluated by the Children’s Global Assessment Scale, deteriorated by an average of six points between 2000 and 2018. Violent threats decreased from 21.5 to 16.6% and violent acts decreased from 26.9 to 20.3%. Suicidal threats decreased from 42.6 to 23.3% in those aged under 13 and remained stable among those aged 13–18. In the 13–18 group, there was an increase in the diagnoses of ADHD, from 5.0 to 16.9% and depression, from 25.1 to 41.7%. However, psychosis decreased from 23.2 to 12.6% in the older age group. In the whole cohort, anxiety disorders increased from 7.6 to 15.6%. The overall picture does not show that CAP inpatients have become more disturbed. While the general functioning of CAP inpatients deteriorated somewhat over the 2000–2018 study period, symptoms of suicidality and violence remained stable or decreased. There was also a continuous increase in short-term treatment.

**Supplementary Information:**

The online version contains supplementary material available at 10.1007/s00787-021-01898-0.

## Introduction

The use of child and adolescent psychiatric (CAP) services has increased worldwide [1–6] and this has led to the expansion of open care services [7, 8]. These include parent training [9, 10], cognitive-behavioral therapy [11, 12], school-based contingency management [13] and pharmacotherapy [14–17]. There are also a growing number of services for those who need more intensive open care treatment, due to the severity of their condition [2, 18], and these include specific multisystemic treatment models [19].

Despite developments to mental health services, children and adolescents with the most severe problems still require inpatient care. Those admitted to hospitals are likely to suffer from high levels of symptom severity and complex psychiatric disorders and they often have multiple psychosocial risk factors and a history of traumatic life events [20–25]. Inpatient admission is often triggered by an acute psychiatric crisis, with a need to stabilize symptoms. These may include suicidality, violent behavior and significant impairment in daily functioning [26–30]. Only a minority of children and adolescents with mental healthcare needs are treated in hospitals, but inpatient treatment accounts for a significant share of the resources allocated to CAP [21, 31, 32]. Most CAP inpatients have been shown to benefit from inpatient care, in terms of functioning level, symptom improvements and stabilization [33].

Only a few studies have assessed time-trends in CAP inpatient treatment and they have reported a trend towards a shorter length of stay (LOS) in hospitals [23, 34, 35] and an increased use of psychotropic medication [14, 15, 36–38]. The present study is based on three cross-sectional studies that used the same methodology to collect data on the CAP inpatient treatment of children and adolescents in 2000, 2011 and 2018. We collected comprehensive nationwide survey data from all CAP wards in Finland during the study years. The 2000 and 2011 data have previously been reported [14, 15, 26, 34, 39–44] and this study extends the findings by another eight years. The same methodology and excellent coverage of the data has given us a unique possibility to examine the changes from 2000 to 2018.

The main aim of this nationwide clinical study was to examine if subjects aged under 13 and 13–18, who were treated in psychiatric inpatient care, had become more severely disturbed during the 2000–2018 study period. This was assessed by general functioning and symptoms of violence and suicidality. The second aim was to study changes in the diagnostic profiles of the patients, namely the hospital LOS, involuntary treatment and recurrent hospitalization.

## Methods

### Subjects and procedure

**Fig. 1:**
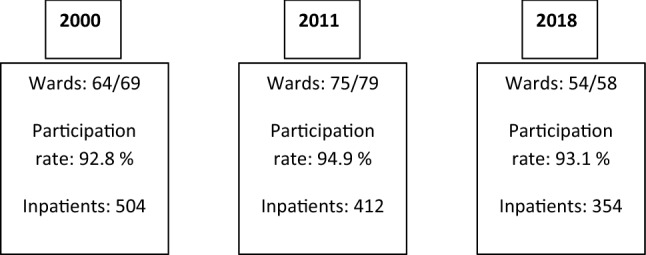
Study flow chart

The nationwide cross-sectional data were collected in 2000, 2011 and 2018 with surveys that explored the background and diagnostic and treatment characteristics of the inpatients. The surveys were sent to all CAP wards in Finland. In Finland, psychiatric inpatients younger than 13 years old are mainly treated in child wards and inpatients from 13 to 17 years in adolescent wards. From the age of 18 years onwards psychiatric inpatients are mainly treated in adult psychiatric wards, which were not part of the data collection. Clinicians responsible for inpatient treatment were asked to fill in a questionnaire for every inpatient who occupied a CAP bed on one chosen study day in each of the study years. Day patients who were treated in hospital wards were classified as inpatients for the purposes of this study. In 2000, there were 69 wards and the data gathered covered 504 inpatients in 64/69 (93%) wards, as five wards declined to take part. In 2011, data were supplied on 412 inpatients from 75/79 (95%) wards and in 2018, we received data on 360 inpatients from 54/58 (93%) wards. Figure 1 shows the study flow chart.

#### Background characteristics

The questionnaire provided data on each patient’s gender and age. It also included their family structure: living with two biological parents, a single mother or father, a cohabiting biological mother or father, or adoptive parents. We also had a category for state care, which was when the child was cared for in a children´s home or foster home.

#### Information on inpatient treatment

LOS in inpatient care was measured as the number of days that each patient had been at the hospital on the specific day when the data were collected. We divided LOS into three categories based on the effect of the changes in the LOS: on general functioning, suicidality and violence, namely 0–30 days, 31–90 days and more than 90 days. Respondents were asked how many times the patients had been hospitalized for psychiatric reasons and whether they were receiving voluntary or involuntary treatment. Involuntary treatment meant treatment against the patient’s will or the will of the parent or caretaker in the case of young children.

#### General functioning

General functioning at the time of the data collection was evaluated with the Children’s Global Assessment Scale (CGAS) [45–47]. This has been designed to reflect the lowest level of functioning of a pediatric patient during a specific time period. The CGAS values range from 1, for the highest level of functional impairment, to 100 for the lowest and the inter-rater reliability has been shown to be moderate [48, 49]. In this study, the CGAS scores were reported as 10-point intervals: 1–10 is presented as one, which represented extremely impaired general functioning, and 91–100 is presented as 10, which represented superior functioning in all areas.

#### Suicidality

The Spectrum of Suicidal Behaviour Scale was used to estimate the suicidality of the inpatients during their ongoing treatment [50, 51]. The scale has been found to have a high inter-rater reliability [52, 53]. The five-point scale covers the following: (1) no suicidal ideation or behavior, (2) suicidal ideas, (3) suicidal threats, (4) mild suicide attempts, and (5) serious suicide attempts. In this study, we divided the responses into the following three groups for the analysis: (1) no suicidality, (2) suicidal threats, including suicidal ideas and suicidal threats, and (3) suicidal attempts, which included mild and serious suicide attempts.

#### Violence

The level of aggression or violence displayed by the inpatients was evaluated with the Spectrum of Assaultive Behaviour Scale [54]. The scale covers the following: (1) no violent ideas or behavior, (2) violent thoughts, (3) violent threats, (4) a less serious violent act, (5) a serious violent act and (6) killing someone. For our analysis, we divided the patients into the following two groups: those scoring 1–3 were categorized as performing no violent acts and those scoring 4–6 were categorized as performing violent acts.

#### Diagnoses

The patients were diagnosed during normal hospital procedures using the International Classification of Diseases, Tenth Revision (ICD-10). The first diagnosis and any possible second diagnoses were included in the analyses. This meant that each patient could appear in more than one diagnostic group. For practical reasons, we have only reported diagnoses given to more than 10 inpatients, or 3% of the total study cohort, in at least one year’s sample. The following diagnostic groups were analyzed: anxiety disorders, attention deficit hyperactivity disorder (ADHD), attachment disorder, autism spectrum disorders, childhood affective disorder, conduct disorder, depression, developmental disorders, eating disorders, mania/bipolar disorder, obsessive compulsive disorder, psychosis and substance abuse.

#### Statistical methods

The differences in the categorical variables between 2000, 2011 and 2018 were calculated with binary logistic regression analysis, with 2018 as the reference year. Odds ratios (OR) and their 95% confidence intervals (95% CI) were calculated to quantify the significant associations. The non-normally distributed LOS variable was analysed using Wilcoxon’s test. Because there were no statistically significant interactions between the study year, gender and diagnoses among the inpatients aged under 13 and 13–18, we combined the data to analyze the gender differences in diagnoses. The differences in general functioning between the years were analyzed using one-way and two-way analysis of variance, with contrasts. P values of less than 0.05 were interpreted as significant. All statistical analyses were carried out using SAS version 9.4 (SAS Institute Inc, Cary, NC, USA).

## Results

### Age, gender and family structure

Table 1 shows the final background characteristics of the 1276 inpatients that we received data on: 504 in 2000, 412 in 2011 and 360 in 2018. The percentage of girls increased from 39.4 in 2000 to 55.3% in 2018, but there were no significant changes in the age distribution. Less than half of the CAP inpatients lived with both biological parents in each study year, and this ranged from 39.3 to 43.7%. Living with a single parent decreased from 34.9 in 2000 to 28.4% in 2018 and living with a biological cohabiting parent increased from 10.4 in 2011 to 16.1% in 2018. During the study period, the percentage of inpatients who were in the state care system doubled, from 5.5 in 2000 to 11.1% in 2018.

**Table 1:** Comparisons of inpatient and treatment characteristics in the study years

	2000	OR (95% CI)	2011	OR (95% CI)	2018
Age (years)		1.2 (0.9–1.5)		1.0 (0.8–1.3)	
1–6	17 (3%)		11 (3%)		8 (2%)
7–12	222 (44%)		158 (40%)		146 (41%)
13–15	149 (30%)		139 (35%)		129 (36%)
16–18	114 (23%)		90 (23%)		77 (21%)
Gender					
Boys	294 (61%)		207 (50%)		161 (45%)
Girls	191 (39%)	0.53 (0.4–0.7)***	205 (50%)	0.8 (0.6–1.1)	199 (55%)
Family structure					
Two biological parents	198 (40%)	1.0 (0.8–1.4)	180 (44%)	1.2 (0.9–1.6)	141 (39%)
Single parent	173 (35%)	1.4 (1.0–1.8)*	131 (32%)	1.2 (0.9–1.6)	102 (28%)
Cohabiting parent	76 (15%)	0.9 (0.7–1.4)	43 (10%)	0.6 (0.4–0.9)*	58 (16%)
Adoptive parents	21 (4%)	0.8 (0.4–1.6)	12 (3%)	0.6 (0.3–1.2)	18 (5%)
State care	27 (5%)	0.5 (0.3–0.8)*	46 (11%)	1.0 (0.6–1.6)	40 (11%)
Previous psychiatric inpatient treatment	186 (38%)	0.7 (0.6–0.9)*	169 (42%)	0.8 (0.6–1.1)	164 (46%)
Involuntary treatment	90 (18%)	0.8 (0.6–1.1)	80 (19%)	0.9 (0.6–1.2)	79 (22%)

Logistic regression analysis was used to examine cumulative (age) or binary (other background variables) with 2018 as the reference year

Bold type indicates statistical significance: *p < 0.05; **p < 0.01; ***p < 0.001

*OR* odds ratio

### Previous psychiatric hospitalization and involuntary treatment

Those who had previously received CAP inpatient treatment before the study rose from 37.6 in 2000 to 45.7% in 2018. More than half (56.2%) were receiving inpatient treatment for the first time, 22.3% for the second time, 10.4% for the third time and 11.0% for four or more times. There were no statistically significant changes in the prevalence of involuntary treatment, which was approximately a fifth (18.2–22.0%) in the study years.

### Length of stay

There was a strong and continuous trend towards shorter median length of stay (LOS). Median LOS fell by three-quarters over the whole study period, from 82.0 days in 2000 to 39 days in 2011 (*p* < 0.001) and then to 20.5 days in 2018 (*p* < 0.001). Mean LOS also decreased, but somewhat less dramatically, from 135.4 days in 2000 to 82.2 days in 2011 (*p* < 0.001) and 55.3 days in 2018 (*p* < 0.001).

### General functioning

The mean general functioning of CAP inpatients continuously decreased. It had fallen to 4.4 in 2018, which equated to 44 in the original CGAS scale, compared 5.0 in 2000 (*p* < 0.001) and 4.7 in 2011 (*p* = 0.008).

We found interactions between gender or age, study year and general functioning: General functioning mainly decreased between 2011 and 2018 in boys (4.8–4.5, *p* = 0.026), but in girls, the decrease occurred between 2000 and 2011 (5.1–4.5, *p* < 0.001). In children aged under 13, general functioning decreased from 4.9 in 2000 to 4.6 in 2018 (*p* = 0.008), with no significant changes between 2011 and 2018. For those aged 13–18, the decreasing trend was continuous from 5.0 in 2000 to 4.6 in 2011 (*p* < 0.001) and 4.3 in 2018 (*p* = 0.017).

**Fig. 2:**
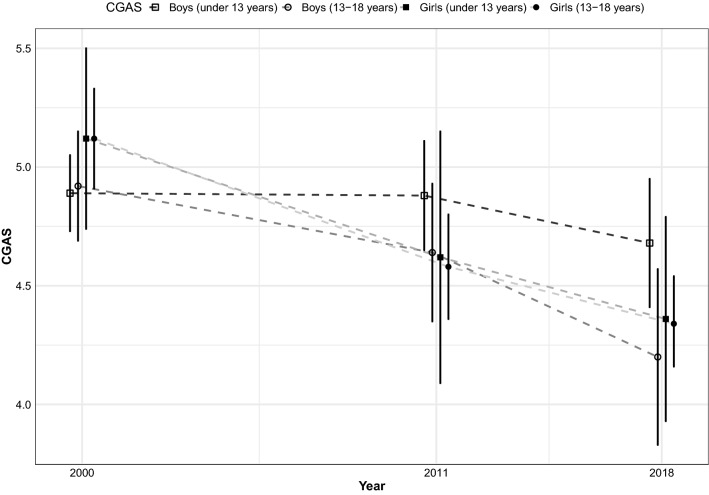
Changes in general functioning, measured with the Children’s Global Assessment Scale, by age and gender

We studied whether there were interactions between LOS, year and general functioning. For the analysis, LOS was divided into 0–30 days, 31–90 days and over 90 days. As described above, there was a significant association between year and general functioning, but adding LOS to the model produced no further interactions (Fig. 2).

### Suicidality

No significant changes were found in suicidal threats or acts from 2000 to 2018.

We found interactions between age, study year and suicidality (Table 2). The prevalence of suicidal threats among inpatients aged under 13 decreased, from 42.6 in 2000 to 38.5% in 2011 to 23.3% in 2018. However, suicidal acts increased in this age group, from 3.0 in 2011 to 11.0% in 2018. Suicidality remained fairly stable in the 13–18 age group, as suicidal threats ranged from 32.7 to 38.3% in the three study years and suicidal acts ranged from 16.2 to 16.9%.

**Table 2:** Suicidality and violence among psychiatric inpatients aged under 13 and 13–18

	2000	OR (95% CI)	2011	OR (95% CI)	2018
Suicidality threats					
All	189 (38%)	1.2 (0.9–1.6)	151 (37%)	1.2 (0.9–1.6)	115 (32%)
Under 13 years	102 (43%)	2.3 (1.4–3.6)***	65 (39%)	1.9 (1.1–3.1)*	36 (23%)
13–18 years	86 (33%)	0.8 (0.5–1.1)	78 (34%)	0.8 (0.5–1.2)	79 (38%)
Suicidality acts					
All	54 (11%)	0.8 (0.5–1.2)	43 (11%)	0.7 (0.5–1.2)	52 (14%)
Under 13 years	10 (4%)	0.5 (0.2–1.1)	5 (3%)	0.3 (0.1–0.9)*	17 (11%)
13–18 years	44 (17%)	0.9 (0.5–1.5)	37 (16%)	0.9 (0.5–1.5)	35 (17%)
Violence threats					
All	108 (22%)	2.0 (1.4–3.0)***	62 (16%)	1.3 (0.8–1.9)	49 (14%)
Boys, under13years	46 (25%)	1.2 (0.6–2.2)	24 (18%)	0.7 (0.4–1.4)	26 (26%)
Boys, 13–18 years	24 (22%)	1.5 (0.6–3.4)	13 (21%)	1.2 (0.5–3.2)	10 (17%)
Girls, under12 years	7 (16%)	2.2 (0.6–8.3)	11 (32%)	7.0 (1.9–25.3)**	4 (7%)
Girls, 13–18 years	24 (17%)	3.3 (1.5–7.3)**	11 (7%)	1.2 (0.5–3.0)	9 (6%)
Violence acts					
All	135 (27%)	1.7 (1.2–2.4)**	96 (24%)	1.3 (0.9–1.9)	73 (21%)
Boys, under13 years	80 (44%)	1.4 (0.8–2.6)	63 (47%)	1.3 (0.7–2.4)	36 (36%)
Boys, 13–18 years	22 (20%)	1.1 (0.5–2.5)	10 (16%)	0.8 (0.3–2.1)	12 (20%)
Girls, under 13 years	7 (16%)	0.7 (0.3–2.1)	7 (21%)	1.5 (0.5–4.5)	12 (22%)
Girls, 13–18 years	21 (15%)	2.0 (0.9–4.1)	13 (9%)	1.0 (0.4–2.2)	13 (9%)

The outcomes that displayed age interactions are shown separately by age groups

The outcomes that displayed age and gender interactions are shown separately for all four patient groups

Binary logistic regression analysis was used with 2018 as the reference years

Bold type indicates statistical significance: *p < 0.05; **p < 0.01; ***p < 0.001

*OR* odds ratio

We also found an interaction between LOS, year and suicidality (*p* = 0.0017). Suicidal threats and acts increased from 2000 to 2018 among inpatients with a LOS of 30 days or less (*p* = 0.0047 and *p* = 0.0074). Suicidal threats decreased from 2000 to 2018 among inpatients with a LOS of 31–90 days (*p* = 0.025). Suicidal threats decreased among inpatients with a LOS of more than 90 days from 2000 to 2018 (*p* = 0.0063) and from 2011 to 2018 (*p* = 0.0126).

### Violence

In 2018, 13.6% of the inpatients had made violent threats and 20.3% had committed violent acts, compared to 21.5% and 26.9%, respectively, in 2000.

We found interactions between gender or age, study year and violence (Table 2). There were no changes in violent threats by boys in both age groups from 2000 to 2018. However, there were significant decreases in violent threats by girls as follows: from 32.3 to 7.3% in those aged 13–18 and 16.6–6.3% in those aged under 13. No significant changes were found in the prevalence of violent acts when they were grouped by age and gender. When the LOS was included in the analysis, the association between the study year and violence disappeared.

### Diagnoses

Table 3 shows the changes in diagnoses. These are primarily shown for the whole cohort but have been split into the two age groups when there are age-related differences. The prevalence of ADHD more than tripled over the study period, from 5.0 in 2000 to 9.8% in 2011 and 16.9% in 2018. Between 2000 and 2018 there were increases in anxiety disorders (7.6–15.6%), childhood affective disorders (8.1–12.2%) and eating disorders (5.0–10%), but there were no significant changes from 2011 to 2018. Substance abuse was rare in all study years and by 2018 it was practically non-existent (0.3%).

**Table 3:** Diagnostic characteristics of the study sample

	2000	OR (95% CI)	2011	OR (95% CI)	2018
Diagnosis					
ADHD	25 (5%)	0.3 (0.2–0.4)***	39 (9%)	0.5 (0.3–0.8)**	61 (17%)
Anxiety disorder	38 (8%)	0.4 (0.3–0.7)***	57 (14%)	0.9 (0.6–1.3)	56 (16%)
Childhood affective disorder	41 (8%)	0.5 (0.3–0.9)*	41 (10%)	0.7 (0.4–1.4)	44 (12%)
Eating disorders	25 (5%)	0.5 (0.3–0.8)**	47 (11%)	1.2 (0.7–1.8)	36 (10%)
Developmental disorder	33 (7%)	1.0 (0.6–1.7)	24 (6%)	0.9 (0.5–1.6)	24 (7%)
Attachment disorder	24 (5%)	1.5 (0.7–3.0)	10 (2%)	0.7 (0.3–1.7)	12 (3%)
Obsessive compulsive disorder	15 (3%)	1.2 (0.5–2.8)	7 (2%)	0.7 (0.3–1.8)	9 (3%)
Mania or bipolar disorder	8 (2%)	0.8 (0.3–2.3)	11 (3%)	1.4 (0.5–3.6)	7 (2%)
Substance abuse	11 (2%)	8.0 (1.0–62.3)*	3 (1%)	2.6 (0.3–25.4)	1 (0%)
Depression					
Under 13 years	34 (14%)	1.1 (0.6–2.0)	16 (9%)	0.7 (0.3–1.4)	20 (13%)
13–18 years	66 (25%)	0.4 (0.3–0.7)***	88 (38%)	0.8 (0.6–1.3)	86 (42%)
Conduct disorder					
Under 13 years	85 (36%)	1.3 (0.8–2.0)	60 (36%)	1.3 (0.8–2.1)	46 (30%)
13–18 years	54 (21%)	3.3 (1.8–6.0)***	18 (8%)	1.1 (0.5–2.2)	15 (7%)
Psychosis					
Under 13 years	7 (3%)	0.9 (0.3–2.9)	9 (5%)	1.7 (0.6–5.1)	5 (3%)
13–18 years	61 (23%)	2.1 (1.3–3.5)**	25 (11%)	0.8 (0.5–1.5)	26 (13%)
Autism spectrum					
Under 13 years	26 (11%)	1.8 (0.8–3.8)	18 (11%)	1.7 (0.8–3.8)	10 (6%)
13–18 years	1 (0%)	0.1 (0.0–0.5)**	9 (4%)	0.7 (0.3–1.6)	12 (6%)

For outcomes with age-interaction the results are shown separately for the two age groups. No gender interactions were found. Binary logistic regression analysis was used with 2018 as the reference year. Only diagnoses with more than 10 inpatients or a 3% share in at least one year’s sample are reported

Bold type indicates statistical significance: *p < 0.05; **p < 0.01; ***p < 0.001

*OR* odds ratio

In the 13–18 age group, there were interactions between age and study year in the prevalence of four diagnoses as follows: depression, autism spectrum disorders, conduct disorder and psychoses. Between 2000 and 2018 there were significant increases in depression (25.1–41.7%) and autism spectrum disorders (0.4–5.8%). In contrast, there were significant decreases in conduct disorders (20.5–7.3%) and psychoses (23.2–12.6%). No changes were found in any of these diagnoses in subjects aged under 13.

## Discussion

A number of key findings emerged from this nationwide, multi-center, cross-sectional, time-trend study on pediatric psychiatric hospital treatment from 2000 to 2018. There was a continuous and substantial shortening of hospital LOS among inpatients, but recurrent hospitalizations increased moderately, and involuntary treatment rates remained stable. There was a moderate deterioration in the general functioning of inpatients and a stable or decreasing trend in suicidality and violence. We also saw significant changes in the distribution of diagnoses. ADHD and anxiety disorders increased in the total cohort, while among subjects aged 13–18 autism spectrum disorders and depression increased, and psychosis and conduct disorders decreased.

### Time trend changes of treatment factors

LOS fell by three-quarters from 2000 to 2018, which reflected previous studies [23, 27, 34, 35]. Changes in the clinical characteristics of the inpatients over the study period are unlikely to explain this major reduction. Non-clinical variables have been reported to be the primary predictors of the LOS in CAP hospitals [55], and this also seemed to be the case in our study. Considerable changes took place in CAP inpatient treatment during the study period. These included a shift from long-term treatment, using techniques like psychodynamic or behavioral frameworks, to medical models that provide acute or crisis treatment. Shorter LOS was accompanied by a parallel increase in the use of psychotropics [15, 35, 36]. Demand for CAP services has increased [1–6] and so have the number of referrals for inpatient care. The number of CAP wards in Finland increased from 69 wards in 2000 to 79 wards in 2011 and then declined to 58 wards in 2018. The marked increase in the number of CAP wards and beds between 2000 and 2011 was driven by both increases in demand of inpatient treatment, and resources allocated to services [15, 56]. The increasing trend in the number of CAP wards has changed during the last 10 years and many of the wards, which participated our data collection in 2011, were by 2018 transformed to provide intensive open care services. The changes in the conditions that CAP wards are facing emphasizes the need for more effective inpatient treatment and prompter discharge. Many hospitalized children and adolescents are in need of enhanced care during and after discharge from the hospital. Models that have been developed for supporting inpatients in the end of hospitalization may enable earlier discharge, reduce readmissions and improve the outcome [57]. Trend towards shorter LOS emphasize the importance of professional skills and adequate staff training. Significantly shorter treatment has made it possible to increase the number of pediatric patients, even with fewer beds.

Shorter LOS probably contributed to the moderate increase in recurrent inpatient treatment between 2000 and 2018, from 38 to 46%, but it was not comparable to the substantially shorter hospital stays. It is possible that trying to reduce LOS has reduced the treatment quality for those who need long psychiatric inpatient stays. Premature discharge could be a reason for recurrent admissions. To prevent this, psychiatric professionals need to work with the patients’ caregivers to improve both the transition from hospital to home and adherence to outpatient care. At the same time, repeated short inpatient treatment may be more beneficial for many young people than a longer hospital stay.

The trend towards growing numbers of inpatient admissions stress the importance of studying and implementing intensive open care service models for children and adolescents with severe psychiatric disorders. These models may provide viable option for inpatient care with similar or possibly superior outcomes [58].

### Time trend changes of patient factors

The CGAS ratings changed significantly from 2000 to 2018 and this indicated lower functioning in more recent years. In general, this deterioration was greater among girls and those aged 13–18. These are novel findings, due to the lack of comprehensive studies on time-trends of general functioning among CAP inpatients. One possible explanation could be that shorter LOS increased the proportion of inpatients with poor general functioning. Previous studies showed that, in general, CGAS ratings improved significantly during inpatient treatment [31, 47, 59]. However, our analysis showed that changes in the LOS did not explain the decrease in general functioning. It is possible that the increased demand for psychiatric treatment has meant that patients with poorer general functioning are more likely to be admitted to CAP wards than those with better functioning. Inpatients with worse general functioning on admission are also likely to shower greater relative improvements [59]. Inpatient care is the most intensive and expensive form of CAP treatment and it has potentially negative effects for some patients [27]. It can be argued that it should mainly be reserved for those with poor general functioning.

Suicidal threats almost halved in subjects under 13, but suicidal acts increased, although the number of cases was relatively small. No changes were found in suicidal symptoms among inpatients aged 13–18. The prevalence of suicidal acts and completed suicides have been shown to increase significantly with age and the onset of puberty [39, 60]. There has been a secular trend towards earlier puberty [61], which may have led to an earlier onset of disorders that are less common among children than adolescents. These include depression [62], which has been associated with suicidality [39, 63]. It is possible that the increases in suicidal acts in subjects aged under 13was partly due to earlier puberty. We also found that suicidal threats and acts increased among inpatients with a LOS of 30 days or less. Suicidality is a major reason for referrals and it often leads to hospital treatment [26, 64]. It is possible that suicidal patients were prioritized due to the high demand for CAP inpatient treatment and the prevalence of suicidality was higher among recent admissions. On the other hand, decreased suicidal thoughts among inpatients with a LOS over 30 days could reflect a movement away from long hospital LOS for this issue and a preference for referrals to intensive open care services, such as dialectical behaviorur therapy [27, 65].

There was a significant decrease in both violent threats and acts from 2000 to 2018 in the total sample. Violent threats particularly decreased in girls. At the same time, the percentage of girls receiving CAP inpatient care increased. This change probably had an effect on the overall trend towards less violence, because boys tend to be more aggressive than girls [66, 67]. Decreasing violence has also been reported in the general Finnish adolescent population [68]. Aggression management practices have been developed [69] and implemented in CAP inpatient wards [70]. There has also been an increase in the use of antipsychotics [71], which may help to decrease violence, because they are often used to tackle aggressive behavior [72]. On the other hand, the high demand for inpatient treatment increases the risk of overcrowded CAP wards, which increases the risk for violence [28, 73]. An interesting finding was that the decreases in violence were associated with shorter LOS. Violent subjects left inpatient treatment sooner in 2018 than in earlier study years. This is in line with previous findings that suggested that aggressive behavior was associated with both shorter LOS and less positive changes in general functioning during CAP inpatient treatment [31]. It seems likely that there has been a change in CAP treatment practices with regard to aggression and violence. These are less frequently considered a reason for CAP hospitalization and the LOS is shorter when inpatient treatment is provided for these issues. Children and adolescents who mainly have externalizing symptoms, such as conduct disorder, are more frequently referred to open care child welfare services and, in severe cases, they are placed in state care [74, 75]. Our study findings on reduced violence agree with the reported decrease in conduct disorders among adolescent CAP inpatients. Conduct disorder has been strongly associated with aggression and is the most common psychiatric disorder in those who enter state care [76, 77].

Our study found that ADHD increased in the whole sample, while autism spectrum disorders only increased among those aged 13–18. During the study period, there were marked increases in these clinical neuropsychiatric diagnoses in the general population of children and adolescents, due to broader diagnostic definitions and increased recognition by professionals [6, 78, 79]. In Finland, awareness of ADHD and autism has increased since the 1990s, due to increased professional training, which has probably contributed to better recognition, increased diagnoses and better treatment of these disorders [80, 81].

We found major increases in affective disorders in subjects aged 13–18, including anxiety disorders, childhood affective disorders and depression. Depression is a major reason for CAP hospital admission [82, 83] and readmission [84] among adolescents. The high prevalence of depression among our older age group may have been partly due to suicidality, which has been strongly associated with both depression [63] and inpatient admissions [26, 64]. The increases in affective disorders were likely to reflect changes at the population level, as they indicated increases in emotional symptoms, especially among those aged 13–18. Accumulating evidence suggests that depressive symptoms have increased in adolescents [85, 86, 88], particularly girls [6]. Once study also provided evidence of an increase in depressive symptoms among 8-year-old Finnish girls [4]. Changes in service provision and diagnostics may contribute to the increase in CAP patients treated for affective disorders, as in neuropsychiatric disorders, but they do not entirely explain it [6].

There were also interesting changes in other diagnoses. In patients aged 13–18, the prevalence of psychosis significantly declined from 2000 to 2011, then remained stable until 2018. This could have been because expanded open care services [87] and increased antipsychotics [7] have reduced the need for inpatient care. Clinicians are now more aware of the possible dissociative and trauma-related mechanisms behind hallucinations [88, 89] and some adolescents may now receive diagnoses of anxiety disorders instead of psychosis. The prevalence of eating disorders increased from 2000 to 2011 and then remained relatively stable, possibly because of changes in treatment. Evidence has been growing that short-term hospital treatment for anorexia nervosa, followed by outpatient care once medical stability has been achieved, is preferable to extended inpatient treatment [90]. Similarly, open care child welfare and CAP services are becoming more popular than inpatient treatment for conduct disorders.

### Time-trend changes in gender and family structure

The percentage of girls continuously increased from 2000 to 2018, in line with increased diagnoses of depression, anxiety disorders and eating disorders, which are more prevalent among girls than boys [34]. An important finding was that CAP inpatients who were in state care doubled from 5.5 to 11% over the study period, compared to the increase from 1.0 to 1.4% observed in the general Finnish population during the same period [91]. This meant that being put in state care was associated with an eight-fold increase in the risk for CAP inpatient treatment. Children and adolescents in state care have been reported to have significantly more psychiatric symptoms than their peers [92]. That was probably the main reason for the major overrepresentation in our study, but it also emphasizes the need to improve lower threshold CAP services for children and adolescents in state care.

### Strengths and limitations

The main strength of this study was the excellent coverage of child and adolescent inpatient psychiatry in Finland, as the data covered 93–95% of the wards in the country 2000, 2011 and 2018. We also used the same methodology for each of the three cross-sectional samples. However, there are significant differences in the CAP services provided by different countries and this restricts the generalizability of the results. The information about the diagnoses was based on clinical evaluations and no systematic structured diagnostic interviews were performed. This reflects clinical reality but may impair the reliability of some diagnoses. Furthermore, although the questionnaire included the detailed description of the CGAS scale, and instructions for its use, no formal training was given to the clinicians and this may have decreased the interrater reliability of the CGAS scores.

## Conclusion

Our study examined nationwide CAP inpatient care in Finland from 200 to 2018. The findings showed that the general functioning of CAP inpatients deteriorated moderately, but the patients were not more disturbed. The dramatic reductions in LOS reflected a more rapid turnover of CAP inpatients, in fewer beds, which meant that proportionally more patients were hospitalized in 2018 than in earlier years. This underlines the need for active treatment strategies and adequate training and skills for acute evaluation and treatment in CAP wards. The changes in diagnostic profiles that we observed, particularly the marked increases in depression and anxiety disorders and the decrease in psychoses, indicate a new role for CAP inpatient treatment. They also stress the importance of close working relationships between inpatient and open care services.

## Supplementary Information

Below is the link to the electronic supplementary material.

Supplementary file1 (DOCX 14 KB)
