# Supplementary material for: Multi-center nationwide study on pediatric psychiatric inpatients 2000–2018: length of stay, recurrent hospitalization, functioning level, suicidality, violence and diagnostic profiles
Source: Eur Child Adolesc Psychiatry. 2021 Nov 22;32(5):835–46. doi: 10.1007/s00787-021-01898-0 (PMC10147780; doi:10.1007/s00787-021-01898-0)
Supplement: Supplementary file 1 — Supplementary file1 (DOCX 14 KB) [file 787_2021_1898_MOESM1_ESM.docx]

**To the supplemental materials**

Diagnostic characteristics. Binary logistic regression analysis year 2018 as a reference year; odds ratios (2000 and 2011 vs 2018) and their confidence intervals. Only diagnoses with over 10 inpatients or 3% share in either 2000 or 2011 are reported. (*p<0.05;**<0.01;***p<0.001)

|  | **Inpatients under 13 years** | | | | | **Inpatients 13-18 years** | | | | |
| --- | --- | --- | --- | --- | --- | --- | --- | --- | --- | --- |
| ***Diagnosis*** | ***2000*** | ***OR(95%Ci)*** | ***2011*** | ***OR(95%Ci)*** | ***2018*** | ***2000*** | ***OR(95%Ci)*** | **2011** | **OR(95%Ci)** | **2018** |
| **Psychosis** | **7(3%)** | **0.9(0.3-2.9)** | **9(5%)** | **1.7(0.6-5.1)** | **5(3%)** | **61(23%)** | **2.1(1.3-3.5)**** | **25(11%)** | **0.8(0.5-1.5)** | **26(13%)** |
| **Substance abuse** | **1(0%)** | **--** | **0(0%)** | **--** | **0/0%)** | **10(4%)** | **8.1(1.0-63.8)*** | **3(1%)** | **2.7(0.3-26.3)** | **1(0%)** |
| **Mania/bipolar disorder** | **0(0%)** | **--** | **3(2%)** | **--** | **0(0%)** | **8(3%)** | **0.9(0.3-2.5)** | **7(3%)** | **0.9(0.3-2.6)** | **7(3%)** |
| **Depression** | **34(14%)** | **1.1(0.6-2.0)** | **16(9%)** | **0.7(0.3-1.4)** | **20(13%)** | **66(25%)** | **0.4(0.3-0.7)***** | **88(38%)** | **0.8(0.6-1.3)** | **86(42%)** |
| **Anxiety disorder** | **16(7%)** | **0.5(0.3-1.1)** | **13(8%)** | **0.6(0.3-1.3)** | **18(12%)** | **22(8%)** | **0.4(0.2-0.7)**** | **43(19%)** | **1.0(0.6-1.7)** | **38(18%)** |
| **Obsessive compulsive disorder** | **7(3%)** | **0.9(0.3-2.9)** | **1(1%)** | **0.2(0.0-1.5)** | **5(3%)** | **8(3%)** | **1.6(0.5-5.3)** | **6(3%)** | **1.4(0.4-4.9)** | **4(2%)** |
| **Eating disorders** | **1(0%)** | **--** | **2(1%)** | **--** | **5(3%)** | **24(9%)** | **0.6(0.3-1.0)*** | **43(19%)** | **1.3(0.8-2.2)** | **31(15%)** |
| **Developmental disorder** | **28(12%)** | **1.1(0.6-2.2)** | **19(11%)** | **1.1(0.5-2.2)** | **16(10%)** | **5(2%)** | **0.5(0.2-1.5)** | **5(2%)** | **0.6(0.2-1.7)** | **8(4%)** |
| **Autism spectrum** | **26(11%)** | **1.8(0.8-3.8)** | **18(11%)** | **1.7(0.8-3.8)** | **10(6%)** | **1(0%)** | **0.1(0.0-0.5)**** | **9(4%)** | **0.7(0.3-1.6)** | **12(6%)** |
| **ADHD** | **19(8%)** | **0.2(0.1-0.4)***** | **32(19%)** | **0.6(0.4-1.1)** | **41(27%)** | **6(2%)** | **0.2(0.1-0.6)**** | **7(3%)** | **0.3(0.1-0.7)**** | **20(10%)** |
| **Conduct disorder** | **85(36%)** | **1.3(0.8-2.0)** | **60(36%)** | **1.3(0.8-2.1)** | **46(30%)** | **54(21%)** | **3.3(1.8-6.0)***** | **18(8%)** | **1.1(0.5-2.2)** | **15(7%)** |
| **Childhood affective disorder** | **20(8%)** | **0.5(0.3-0.9)*** | **21(12%)** | **0.7(0.4-1.4)** | **25(16%)** | **21(8%)** | **0.9(0.5-1.6)** | **20(9%)** | **0.9(0.5-1.8)** | **19(9%)** |
| **Attachment disorder** | **22(9%)** | **1.3(0.6-2.8)** | **9(5%)** | **0.7(0.3-1.8)** | **11(7%)** | **2(1%)** | **1.6(0.1-17.4)** | **1(0%)** | **0.9(0.1-14.5** | **1(1%)** |
